# 5-Bromo-3-ethyl­sulfinyl-7-methyl-2-(4-methyl­phen­yl)-1-benzo­furan

**DOI:** 10.1107/S1600536814005844

**Published:** 2014-03-22

**Authors:** Hong Dae Choi, Pil Ja Seo, Uk Lee

**Affiliations:** a Department of Chemistry, Dongeui University, San 24 Kaya-dong, Busanjin-gu, Busan 614-714, Republic of Korea; b Department of Chemistry, Pukyong National University, 599-1 Daeyeon 3-dong, Nam-gu, Busan 608-737, Republic of Korea

## Abstract

In the title compound, C_18_H_17_BrO_2_S, the dihedral angle between the mean planes of the benzo­furan and 4-methyl­phenyl rings is 14.54 (5)°. In the crystal, mol­ecules are linked *via* pairs of π–π inter­actions between the benzene and 4-methyl­phenyl rings, with centroid–centroid distances of 3.811 (3) and 3.755 (3) Å. A similar inter­action is found between the furan and 4-methyl­phenyl rings, with a centroid–centroid distance of 3.866 (3) Å between neighbouring mol­ecules. The mol­ecules are stacked along the *a*-axis direction. In addition, a short Br⋯O contact distance of 3.128 (2) Å is observed between inversion-related dimers.

## Related literature   

For background information and the crystal structures of related compounds, see: Choi *et al.* (2010*a* ▶,*b* ▶). For a review of halogen bonding, see: Politzer *et al.* (2007 ▶). For π–π stacking in metal complexes with aromatic nitro­gen ligands, see: Janiak (2000 ▶).
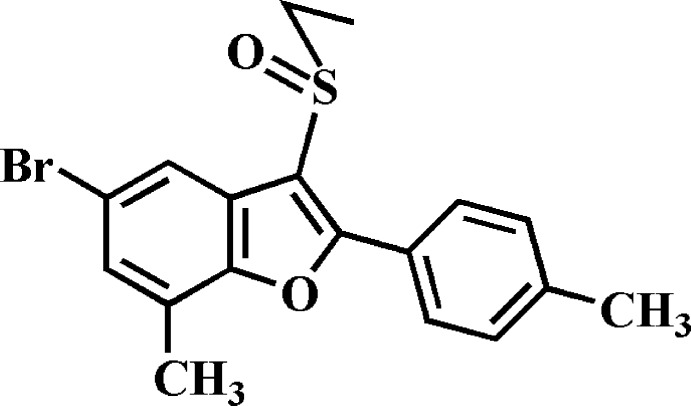


## Experimental   

### 

#### Crystal data  

C_18_H_17_BrO_2_S
*M* *_r_* = 377.29
Triclinic, 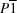

*a* = 7.3921 (2) Å
*b* = 10.2909 (3) Å
*c* = 11.8701 (3) Å
α = 68.867 (1)°
β = 89.146 (1)°
γ = 71.361 (1)°
*V* = 792.91 (4) Å^3^
*Z* = 2
Mo *K*α radiation
μ = 2.73 mm^−1^
*T* = 173 K
0.37 × 0.25 × 0.22 mm

#### Data collection  

Bruker SMART APEXII CCD diffractometer
Absorption correction: multi-scan (*SADABS*; Bruker, 2009 ▶) *T*_min_ = 0.535, *T*_max_ = 0.746
13645 measured reflections
3457 independent reflections
3145 reflections with *I* > 2σ(*I*)
*R*_int_ = 0.035

#### Refinement  

*R*[*F* ^2^ > 2σ(*F* ^2^)] = 0.029
*wR*(*F* ^2^) = 0.076
*S* = 1.04
3457 reflections
202 parameters
H-atom parameters constrained
Δρ_max_ = 0.66 e Å^−3^
Δρ_min_ = −0.47 e Å^−3^

### 

Data collection: *APEX2* (Bruker, 2009 ▶); cell refinement: *SAINT* (Bruker, 2009 ▶); data reduction: *SAINT*; program(s) used to solve structure: *SHELXS97* (Sheldrick, 2008 ▶); program(s) used to refine structure: *SHELXL97* (Sheldrick, 2008 ▶); molecular graphics: *ORTEP-3 for Windows* (Farrugia, 2012 ▶) and *DIAMOND* (Brandenburg, 1998 ▶); software used to prepare material for publication: *SHELXL97*.

## Supplementary Material

Crystal structure: contains datablock(s) I. DOI: 10.1107/S1600536814005844/fj2667sup1.cif

Structure factors: contains datablock(s) I. DOI: 10.1107/S1600536814005844/fj2667Isup2.hkl

Supporting information file. DOI: 10.1107/S1600536814005844/fj2667Isup3.cml

CCDC reference: 992016

Additional supporting information:  crystallographic information; 3D view; checkCIF report

## Figures and Tables

**Fig. 1.**
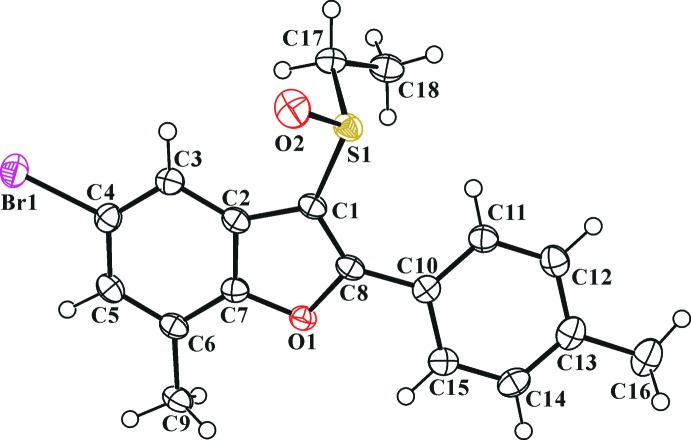
The molecular structure of the title molecule with the atom numbering scheme. The displacement ellipsoids are drawn at the 50% probability level. The hydrogen atoms are presented as small spheres of arbitrary radius.

**Fig. 2.**
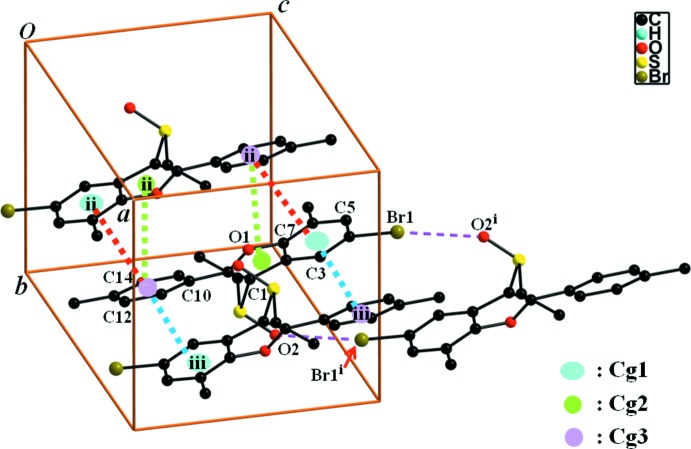
A view of the Br···O and π–π interactions (dotted lines) in the crystal structure of the title compound. The H-atoms were omitted for clarity. [Symmetry codes: (i)- x + 2, - y + 1, - z + 2; (ii) -x + 1, -y + 1, -z + 1; (iii) -x + 2, -y + 1, -z + 1.]

## supplementary crystallographic information

### 1. Comment

As a part of our continuing study of 5-bromo-3-ethylsulfinyl-7-methyl-1-benzofuran derivatives containing 4-fluorophenyl (Choi *et al.*, 2010*a*) and 4-chlorophenyl (Choi *et al.*, 2010*b*) substituents in 2-position, we report here on the crystal structure of the title compound.

In the title molecule (Fig. 1), the benzofuran ring system is essentially planar, with a mean deviation of 0.017 (2) Å from the least-squares plane defined by the nine constituent atoms. The 4-methylphenyl ring is essentially planar, with a mean deviation of 0.004 (1) Å from the least-squares plane defined by the six constituent atoms. The dihedral angle formed by the benzofuran ring system and the 4-methylphenyl ring is 14.54 (5)°.

In the crystal structure (Fig. 2), the molecules are linked *via*π–π interactions between the benzene and 4-methylphenyl rings, and the furan and 4-methylphenyl rings of neighbouring molecules. The molecules stack along the *a*-axis direction. The relevant centroid names of π–π stacking interactions are Cg1 for the benzene ring (C2–C7), Cg2 for the furan ring (C1/C2/C7/O1/O8) and Cg3 for the 4-methylphenyl ring (C10–C15). The centroid–centroid separations of Cg1···Cg3^ii^, Cg1···Cg3^iii^ and Cg2···Cg3^ii^ are 3.811 (2), 3.755 (2) and 3.866 (2) Å, respectively. The symmetry codes are:(ii) -*x* + 1, -*y* + 1, -*z* + 1; (iii)-*x* + 2, -*y* + 1, -*z* + 1. Both interplanar angles between the rings (C2–C7) and (C10–C15)^ii^ as well as (C2–C7) and (C10–C15)^iii^, and (C1/C2/C7/O1/C8) and (C10–C15)^ii^ equal to 13.90 (5)° and 15.66 (5)°, respectively. This angle is quite large for rings being in π-electron···π-electron interactions as it follows from the study by Janiak (2000) who investigated π–π stacking in metal complexes with aromatic nitrogen ligands. According to Fig. 8 of Janiak's study, the interplanar angles between the rings that are involved in π-electron···π-electron interactions are less than 10° in overwhelming majority of cases. In addition, Br···O halogen-bondings (Politzer *et al.*, 2007) between the bromine atom and the oxygen atom of the S═O unit [Br1···O2^i^ = 3.128 (2) Å C4—Br1···O2^i^ = 164.97 (7)°, (symmetry code :(i) - *x* + 2, - *y* + 1, - *z* + 2)] are observed between inversion-related dimers.

### 2. Experimental

3-Chloroperoxybenzoic acid (77%, 224 mg, 1.0 mmol) was added in small portions to a stirred solution of 5-bromo-3-ethylsulfanyl-7-methyl-2-(4-methylphenyl)-1-benzofuran (361 mg, 0.9 mmol) in dichloromethane (25 mL) at 273 K. After being stirred at room temperature for 5h, the mixture was washed with saturated sodium bicarbonate solution and the organic layer was separated, dried over magnesium sulfate, filtered and concentrated at reduced pressure. The residue was purified by column chromatography (hexane–ethyl acetate, 1:1 *v*/*v*) to afford the title compound as a colorless solid [yield 71%, m.p. 419–420 K; *R*_f_ = 0.51 (hexane-ethyl acetate, 1:1 *v*/*v*)]. Single crystals suitable for X-ray diffraction were prepared by slow evaporation of a solution of the title compound in ethyl acetate at room temperature.

### 3. Refinement

All H atoms were positioned geometrically and refined using a riding model, with C—H = 0.95 Å for aryl, 0.99 Å for methylene and 0.98 Å for methyl H atoms, respectively. *U*_iso_ (H) = 1.2*U*_eq_ (C) for aryl and methylene, and 1.5*U*_eq_ (C) for methyl H atoms. The positions of methyl hydrogens were optimized using the *SHELXL*-97's command AFIX 137 (Sheldrick, 2008).

### Crystal data

**Table:**

C_18_H_17_BrO_2_S	*Z* = 2
*M**_r_* = 377.29	*F*(000) = 384
Triclinic, *P*1	*D*_x_ = 1.580 Mg m^−^^3^
Hall symbol: -P 1	Melting point = 420–419 K
*a* = 7.3921 (2) Å	Mo *K*α radiation, λ = 0.71073 Å
*b* = 10.2909 (3) Å	Cell parameters from 8443 reflections
*c* = 11.8701 (3) Å	θ = 2.3–28.3°
α = 68.867 (1)°	µ = 2.73 mm^−^^1^
β = 89.146 (1)°	*T* = 173 K
γ = 71.361 (1)°	Block, colourless
*V* = 792.91 (4) Å^3^	0.37 × 0.25 × 0.22 mm

### Data collection

**Table:**

Bruker SMART APEXII CCD diffractometer	3457 independent reflections
Radiation source: rotating anode	3145 reflections with *I* > 2σ(*I*)
Graphite multilayer monochromator	*R*_int_ = 0.035
Detector resolution: 10.0 pixels mm^-1^	θ_max_ = 27.0°, θ_min_ = 1.9°
φ and ω scans	*h* = −8→9
Absorption correction: multi-scan (*SADABS*; Bruker, 2009)	*k* = −11→13
*T*_min_ = 0.535, *T*_max_ = 0.746	*l* = −15→15
13645 measured reflections	

### Refinement

**Table:**

Refinement on *F*^2^	Primary atom site location: structure-invariant direct methods
Least-squares matrix: full	Secondary atom site location: difference Fourier map
*R*[*F*^2^ > 2σ(*F*^2^)] = 0.029	Hydrogen site location: difference Fourier map
*wR*(*F*^2^) = 0.076	H-atom parameters constrained
*S* = 1.04	*w* = 1/[σ^2^(*F*_o_^2^) + (0.0422*P*)^2^ + 0.3263*P*] where *P* = (*F*_o_^2^ + 2*F*_c_^2^)/3
3457 reflections	(Δ/σ)_max_ = 0.001
202 parameters	Δρ_max_ = 0.66 e Å^−^^3^
0 restraints	Δρ_min_ = −0.47 e Å^−^^3^

### Special details

**Table:**

Geometry. All esds (except the esd in the dihedral angle between two l.s. planes) are estimated using the full covariance matrix. The cell esds are taken into account individually in the estimation of esds in distances, angles and torsion angles; correlations between esds in cell parameters are only used when they are defined by crystal symmetry. An approximate (isotropic) treatment of cell esds is used for estimating esds involving l.s. planes.
Refinement. Refinement of F^2^ against ALL reflections. The weighted R-factor wR and goodness of fit S are based on F^2^, conventional R-factors R are based on F, with F set to zero for negative F^2^. The threshold expression of F^2^ > 2sigma(F^2^) is used only for calculating R-factors(gt) etc. and is not relevant to the choice of reflections for refinement. R-factors based on F^2^ are statistically about twice as large as those based on F, and R- factors based on ALL data will be even larger.

### Fractional atomic coordinates and isotropic or equivalent isotropic displacement parameters (Å^2^)

**Table:**

	*x*	*y*	*z*	*U*_iso_*/*U*_eq_	
Br1	0.99213 (3)	0.28117 (2)	1.076443 (17)	0.03316 (9)	
S1	0.64111 (7)	0.80626 (5)	0.58929 (4)	0.02572 (12)	
O1	0.80567 (19)	0.41973 (14)	0.55125 (12)	0.0243 (3)	
O2	0.7922 (2)	0.81341 (17)	0.66688 (15)	0.0380 (4)	
C1	0.7074 (3)	0.6207 (2)	0.60113 (17)	0.0226 (4)	
C2	0.7941 (3)	0.4927 (2)	0.71109 (17)	0.0232 (4)	
C3	0.8306 (3)	0.4680 (2)	0.83342 (17)	0.0251 (4)	
H3	0.7908	0.5463	0.8627	0.030*	
C4	0.9278 (3)	0.3240 (2)	0.90957 (17)	0.0262 (4)	
C5	0.9886 (3)	0.2066 (2)	0.86941 (18)	0.0269 (4)	
H5	1.0556	0.1097	0.9262	0.032*	
C6	0.9532 (3)	0.2284 (2)	0.74878 (18)	0.0252 (4)	
C7	0.8532 (3)	0.3738 (2)	0.67370 (17)	0.0231 (4)	
C8	0.7173 (3)	0.5711 (2)	0.50770 (17)	0.0231 (4)	
C9	1.0208 (3)	0.1073 (2)	0.70051 (19)	0.0314 (4)	
H9A	0.9108	0.0820	0.6816	0.047*	
H9B	1.1140	0.0198	0.7618	0.047*	
H9C	1.0816	0.1406	0.6266	0.047*	
C10	0.6536 (3)	0.6380 (2)	0.37788 (17)	0.0236 (4)	
C11	0.5193 (3)	0.7799 (2)	0.32352 (18)	0.0273 (4)	
H11	0.4689	0.8370	0.3715	0.033*	
C12	0.4589 (3)	0.8382 (2)	0.20024 (18)	0.0302 (4)	
H12	0.3686	0.9355	0.1646	0.036*	
C13	0.5276 (3)	0.7571 (2)	0.12746 (18)	0.0305 (4)	
C14	0.6616 (3)	0.6152 (2)	0.18236 (19)	0.0309 (4)	
H14	0.7102	0.5578	0.1345	0.037*	
C15	0.7248 (3)	0.5567 (2)	0.30458 (18)	0.0273 (4)	
H15	0.8175	0.4604	0.3395	0.033*	
C16	0.4606 (3)	0.8183 (3)	−0.00608 (19)	0.0407 (5)	
H16A	0.3793	0.7662	−0.0213	0.061*	
H16B	0.5721	0.8047	−0.0517	0.061*	
H16C	0.3865	0.9241	−0.0324	0.061*	
C17	0.4326 (3)	0.8128 (2)	0.67180 (19)	0.0314 (5)	
H17A	0.4619	0.7210	0.7452	0.038*	
H17B	0.4015	0.8974	0.6986	0.038*	
C18	0.2603 (3)	0.8292 (2)	0.5931 (2)	0.0336 (5)	
H18A	0.2232	0.9248	0.5247	0.050*	
H18B	0.1527	0.8241	0.6418	0.050*	
H18C	0.2939	0.7492	0.5621	0.050*	

### Atomic displacement parameters (Å^2^)

**Table:**

	*U*^11^	*U*^22^	*U*^33^	*U*^12^	*U*^13^	*U*^23^
Br1	0.03870 (14)	0.03266 (13)	0.02694 (12)	−0.01276 (9)	−0.00261 (8)	−0.00897 (9)
S1	0.0267 (2)	0.0211 (2)	0.0298 (2)	−0.00751 (18)	0.00028 (19)	−0.01041 (18)
O1	0.0245 (7)	0.0218 (6)	0.0263 (6)	−0.0059 (5)	0.0029 (5)	−0.0104 (5)
O2	0.0368 (9)	0.0364 (8)	0.0449 (9)	−0.0163 (7)	−0.0060 (7)	−0.0162 (7)
C1	0.0182 (9)	0.0212 (9)	0.0288 (9)	−0.0062 (7)	0.0028 (7)	−0.0103 (7)
C2	0.0171 (9)	0.0233 (9)	0.0291 (9)	−0.0067 (7)	0.0035 (7)	−0.0098 (7)
C3	0.0231 (9)	0.0256 (9)	0.0287 (9)	−0.0088 (7)	0.0026 (7)	−0.0122 (8)
C4	0.0240 (10)	0.0307 (10)	0.0258 (9)	−0.0109 (8)	0.0015 (7)	−0.0111 (8)
C5	0.0211 (9)	0.0237 (9)	0.0331 (10)	−0.0070 (7)	0.0004 (8)	−0.0079 (8)
C6	0.0184 (9)	0.0244 (9)	0.0332 (10)	−0.0067 (7)	0.0031 (7)	−0.0118 (8)
C7	0.0194 (9)	0.0250 (9)	0.0273 (9)	−0.0083 (7)	0.0038 (7)	−0.0118 (7)
C8	0.0173 (9)	0.0212 (9)	0.0308 (9)	−0.0064 (7)	0.0045 (7)	−0.0101 (7)
C9	0.0299 (11)	0.0227 (9)	0.0382 (11)	−0.0026 (8)	0.0025 (8)	−0.0129 (8)
C10	0.0212 (9)	0.0274 (9)	0.0260 (9)	−0.0123 (7)	0.0048 (7)	−0.0107 (7)
C11	0.0239 (10)	0.0287 (10)	0.0299 (10)	−0.0085 (8)	0.0045 (8)	−0.0122 (8)
C12	0.0222 (10)	0.0313 (10)	0.0327 (10)	−0.0086 (8)	0.0020 (8)	−0.0076 (8)
C13	0.0260 (10)	0.0408 (12)	0.0272 (10)	−0.0178 (9)	0.0049 (8)	−0.0102 (8)
C14	0.0320 (11)	0.0383 (11)	0.0311 (10)	−0.0180 (9)	0.0102 (8)	−0.0176 (9)
C15	0.0279 (10)	0.0259 (9)	0.0310 (10)	−0.0118 (8)	0.0069 (8)	−0.0118 (8)
C16	0.0367 (12)	0.0562 (15)	0.0283 (10)	−0.0197 (11)	0.0034 (9)	−0.0112 (10)
C17	0.0298 (11)	0.0311 (10)	0.0316 (10)	−0.0036 (8)	0.0054 (8)	−0.0156 (8)
C18	0.0290 (11)	0.0328 (11)	0.0440 (12)	−0.0119 (9)	0.0085 (9)	−0.0189 (9)

### Geometric parameters (Å, º)

**Table:**

Br1—C4	1.8996 (19)	C9—H9C	0.9800
Br1—O2^i^	3.1284 (15)	C10—C11	1.396 (3)
S1—O2	1.4943 (15)	C10—C15	1.401 (3)
S1—C1	1.7658 (19)	C11—C12	1.384 (3)
S1—C17	1.810 (2)	C11—H11	0.9500
O1—C7	1.369 (2)	C12—C13	1.392 (3)
O1—C8	1.378 (2)	C12—H12	0.9500
C1—C8	1.370 (3)	C13—C14	1.395 (3)
C1—C2	1.447 (3)	C13—C16	1.503 (3)
C2—C7	1.389 (3)	C14—C15	1.377 (3)
C2—C3	1.396 (3)	C14—H14	0.9500
C3—C4	1.379 (3)	C15—H15	0.9500
C3—H3	0.9500	C16—H16A	0.9800
C4—C5	1.397 (3)	C16—H16B	0.9800
C5—C6	1.382 (3)	C16—H16C	0.9800
C5—H5	0.9500	C17—C18	1.519 (3)
C6—C7	1.390 (3)	C17—H17A	0.9900
C6—C9	1.496 (3)	C17—H17B	0.9900
C8—C10	1.456 (3)	C18—H18A	0.9800
C9—H9A	0.9800	C18—H18B	0.9800
C9—H9B	0.9800	C18—H18C	0.9800
			
C4—Br1—O2^i^	164.97 (7)	C11—C10—C8	122.23 (17)
O2—S1—C1	106.64 (9)	C15—C10—C8	119.43 (17)
O2—S1—C17	106.82 (10)	C12—C11—C10	120.49 (18)
C1—S1—C17	98.32 (10)	C12—C11—H11	119.8
C7—O1—C8	107.13 (14)	C10—C11—H11	119.8
C8—C1—C2	107.24 (16)	C11—C12—C13	121.33 (19)
C8—C1—S1	127.11 (15)	C11—C12—H12	119.3
C2—C1—S1	124.84 (14)	C13—C12—H12	119.3
C7—C2—C3	119.30 (17)	C12—C13—C14	117.90 (18)
C7—C2—C1	104.87 (16)	C12—C13—C16	121.8 (2)
C3—C2—C1	135.80 (17)	C14—C13—C16	120.3 (2)
C4—C3—C2	116.46 (17)	C15—C14—C13	121.33 (19)
C4—C3—H3	121.8	C15—C14—H14	119.3
C2—C3—H3	121.8	C13—C14—H14	119.3
C3—C4—C5	123.18 (18)	C14—C15—C10	120.62 (18)
C3—C4—Br1	119.36 (15)	C14—C15—H15	119.7
C5—C4—Br1	117.42 (14)	C10—C15—H15	119.7
C6—C5—C4	121.36 (17)	C13—C16—H16A	109.5
C6—C5—H5	119.3	C13—C16—H16B	109.5
C4—C5—H5	119.3	H16A—C16—H16B	109.5
C5—C6—C7	114.64 (17)	C13—C16—H16C	109.5
C5—C6—C9	123.41 (17)	H16A—C16—H16C	109.5
C7—C6—C9	121.92 (18)	H16B—C16—H16C	109.5
O1—C7—C2	110.82 (16)	C18—C17—S1	111.08 (15)
O1—C7—C6	124.11 (16)	C18—C17—H17A	109.4
C2—C7—C6	125.04 (18)	S1—C17—H17A	109.4
C1—C8—O1	109.93 (16)	C18—C17—H17B	109.4
C1—C8—C10	135.84 (17)	S1—C17—H17B	109.4
O1—C8—C10	114.21 (16)	H17A—C17—H17B	108.0
C6—C9—H9A	109.5	C17—C18—H18A	109.5
C6—C9—H9B	109.5	C17—C18—H18B	109.5
H9A—C9—H9B	109.5	H18A—C18—H18B	109.5
C6—C9—H9C	109.5	C17—C18—H18C	109.5
H9A—C9—H9C	109.5	H18A—C18—H18C	109.5
H9B—C9—H9C	109.5	H18B—C18—H18C	109.5
C11—C10—C15	118.32 (17)		
			
O2—S1—C1—C8	−130.72 (18)	C5—C6—C7—C2	1.6 (3)
C17—S1—C1—C8	118.85 (18)	C9—C6—C7—C2	−176.86 (19)
O2—S1—C1—C2	37.58 (19)	C2—C1—C8—O1	−0.3 (2)
C17—S1—C1—C2	−72.85 (18)	S1—C1—C8—O1	169.66 (13)
C8—C1—C2—C7	0.9 (2)	C2—C1—C8—C10	177.6 (2)
S1—C1—C2—C7	−169.35 (14)	S1—C1—C8—C10	−12.4 (3)
C8—C1—C2—C3	179.1 (2)	C7—O1—C8—C1	−0.4 (2)
S1—C1—C2—C3	8.8 (3)	C7—O1—C8—C10	−178.87 (16)
C7—C2—C3—C4	1.0 (3)	C1—C8—C10—C11	−15.2 (4)
C1—C2—C3—C4	−177.0 (2)	O1—C8—C10—C11	162.70 (17)
C2—C3—C4—C5	0.1 (3)	C1—C8—C10—C15	166.5 (2)
C2—C3—C4—Br1	177.69 (14)	O1—C8—C10—C15	−15.6 (3)
C3—C4—C5—C6	−0.4 (3)	C15—C10—C11—C12	−0.1 (3)
Br1—C4—C5—C6	−178.01 (15)	C8—C10—C11—C12	−178.39 (19)
C4—C5—C6—C7	−0.4 (3)	C10—C11—C12—C13	0.8 (3)
C4—C5—C6—C9	177.98 (19)	C11—C12—C13—C14	−0.7 (3)
C8—O1—C7—C2	1.0 (2)	C11—C12—C13—C16	179.0 (2)
C8—O1—C7—C6	−176.82 (18)	C12—C13—C14—C15	−0.2 (3)
C3—C2—C7—O1	−179.73 (16)	C16—C13—C14—C15	−179.9 (2)
C1—C2—C7—O1	−1.2 (2)	C13—C14—C15—C10	0.9 (3)
C3—C2—C7—C6	−1.9 (3)	C11—C10—C15—C14	−0.8 (3)
C1—C2—C7—C6	176.64 (18)	C8—C10—C15—C14	177.56 (18)
C5—C6—C7—O1	179.12 (17)	O2—S1—C17—C18	171.58 (14)
C9—C6—C7—O1	0.7 (3)	C1—S1—C17—C18	−78.14 (16)

Symmetry code: (i) −*x*+2, −*y*+1, −*z*+2.
